# Correlation between breast cancer subtypes determined by immunohistochemistry and n-COUNTER PAM50 assay: a real-world study

**DOI:** 10.1007/s10549-023-07094-9

**Published:** 2023-09-29

**Authors:** Sara Lopez-Tarruella, María Del Monte-Millán, Marta Roche-Molina, Yolanda Jerez, Isabel Echavarria Diaz-Guardamino, Blanca Herrero López, Salvador Gamez Casado, Iván Marquez-Rodas, Enrique Alvarez, María Cebollero, Tatiana Massarrah, Inmaculada Ocaña, Ainhoa Arias, José Ángel García-Sáenz, Fernando Moreno Anton, Clara Olier Garate, Diana Moreno Muñoz, David Marrupe, Miguel Ángel Lara Álvarez, Santos Enrech, Coralia Bueno Muiño, Miguel Martín

**Affiliations:** 1 Medical Oncology Department, Hospital General Universitario Gregorio Marañón, Instituto de Investigación Sanitaria Gregorio Marañon (IiSGM), CIBERONC, Geicam, Universidad Complutense, 28007 Madrid, Spain; 2 Medical Oncology Department, Hospital General Universitario Gregorio Marañón, Instituto de Investigación Sanitaria Gregorio Marañón (IiSGM), CiberOnc, Madrid, Spain; 3 Medical Oncology Department, Hospital General Universitario Gregorio Marañón Instituto de Investigación Sanitaria Gregorio Marañón (IiSGM), Madrid, Spain; 4 Pathology Service, Hospital General Universitario Gregorio Marañón, Madrid, Spain; 5 Medical Oncology Department, Hospital Clínico San Carlos, Instituto de Investigación Sanitaria San Carlos (IdISSC), CIBERONC, Madrid, Spain; 6 Medical Oncology Department, Hospital Universitario Fundación Alcorcón, Alcorcon, Spain; 7 Department of Oncologia, Hospital Universitario de Móstoles, Mostoles, Spain; 8 Medical Oncology Department, Hospital Universitario Infanta Leonor, Universidad Complutense, Madrid, Spain; 9 Medical Oncology Department, Hospital Universitario de Getafe, Madrid, Spain; 10 Medical Oncology Department, Hospital Infanta Cristina (Parla), Fundación de Investigación Biomédica del H.U. Puerta de Hierro, Majadahonda, 28009 Madrid, Spain

**Keywords:** Breast cancer, Gene expression profiling, Prognostic platforms, Biomarkers

## Abstract

**Purpose:**

Molecular subtyping based on gene expression profiling (i.e., PAM50 assay) aids in determining the prognosis and treatment of breast cancer (BC), particularly in hormone receptor (HR)-positive/human epidermal growth factor receptor 2 (HER2)-negative tumors, where luminal A and B subtypes have different prognoses and treatments. Several surrogate classifications have been proposed for distinguishing between the luminal A and B subtypes. This study determines the accuracy of local immunohistochemistry (IHC) techniques for classifying HR-positive/HER2-negative (HR+/HER2−) tumors according to intrinsic subtypes using the nCOUNTER PAM50 assay as reference and the HR status definition according the ASCO/CAP recommendations.

**Methods:**

Molecular subtypes resulting from nCOUNTER PAM50 performed in our laboratory between 2014 and 2020 were correlated with three different proxy surrogates proposed in the literature based on ER, PR, HER2, and Ki67 expression with different cut-off values. Concordance was measured using the level of agreement and kappa statistics.

**Results:**

From 1049 samples with the nCOUNTER test, 679 and 350 were luminal A and B subtypes, respectively. Only a poor-to-fair correlation was observed between the three proxy surrogates and real genomic subtypes as determined by nCOUNTER PAM50. Moreover, 5–11% and 18–36% of the nCOUNTER PAM50 luminal B and A tumors were classified as luminal A and B, respectively, by these surrogates.

**Conclusion:**

The concordance between luminal subtypes determined by three different IHC-based classifiers and the nCOUNTER PAM50 assay was suboptimal. Thus, a significant proportion of luminal A and B tumors as determined by the surrogate classifiers could be undertreated or over-treated.

**Supplementary Information:**

The online version contains supplementary material available at 10.1007/s10549-023-07094-9.

## Introduction

Breast cancer (BC), which is a biologically heterogeneous disease, has been classified into different subgroups according to its biological characteristics that correlate with varying clinical behavior patterns and responses to therapy. Traditionally, according to the pathologic criteria, BC has been broadly grouped into three categories based on the expression of hormone receptors (estrogen and progesterone) (HR) and human epidermal growth factor receptor 2 (HER2). The first taxonomic classification of BC was proposed in the beginning of the twenty-first century by Perou et al., who identified different molecular entities carrying different prognoses using gene expression profiling [1]. The intrinsic subtype classification defined four tumor subtypes, namely luminal A, luminal B, HER2 enriched, and basal like, with unique biological characteristics, that represent a paradigm shift in understanding the biology of BC considering important clinical implications at different levels. One of the most important translational values, provided by this classification system has been its application in the selection of patients with HR+/HER2− tumors that are candidates for adjuvant systemic chemotherapy based on the estimation of the prognosis. Essentially, luminal B tumors are more proliferative, have a worse prognosis, and benefit more from the addition of adjuvant chemotherapy to endocrine therapy than the luminal A subtype, which can be treated solely with adjuvant endocrine therapy with excellent outcomes. The Cancer Genome Atlas (TCGA), carried out in a series of more than 400 human BC samples, integrates data from genomic DNA copy number arrays, DNA methylation analysis, exome sequencing, messenger RNA arrays, microRNA sequencing, and reverse-phase protein arrays, confirming the consistency of the four intrinsic subtype classifications by Perou et al. [1] and enriching the biological perspective of these major subtypes of BC [2]. Therefore, luminal subtype (A or B) identification could be of great help in the selection of adjuvant therapy beyond previous tools that were built to estimate survival mainly based on the clinicopathological features (i.e., Nottingham prognostic index or Adjuvant Online) and later evolved to online prognostic calculators, including more clinically relevant information in the model, but still missed crucial biological attributes (i.e., CancerMath or PREDICT).

Intrinsic subtype classification of BC has been demonstrated to be reproducible and potentially useful for estimating prognosis and predicting treatment response in numerous studies. Therefore, the next step was developing a standardized assay for implementation in a clinical setting. Parker et al. reproducibly established four main intrinsic subtypes by supervised clustering of genome-wide mRNA expression data and designed a polymerase chain reaction test based on 50 genes (then called the PAM50 signature), which could be performed in archival samples and added prognostic and predictive value to the standard clinicopathological markers [3]. Recently, NanoString nCOUNTER technology was introduced as a fast and reliable method for establishing intrinsic subtypes in a single hybridization process that does not require enzymes [4].

Since gene expression analysis is expensive and not universally feasible, several surrogate classifications based on immunohistochemistry and/or *in situ* hybridization standard techniques (estrogen receptor [ER], progesterone receptor [PR], Ki67, and HER2 expression) have been proposed [5–7] to guide BC treatment algorithms in clinical practice setting [8–10]. In this study, we primarily analyzed the correlation between three proposed surrogate molecular classifications [5–7] and the Prosigna^®^ assay to evaluate their accuracy in predicting the intrinsic subtype in a real-world early BC setting. Furthermore, upon focusing on the luminal population, we aimed to identify the Ki67 cut-off that would help differentiate between the luminal A and B subtypes more accurately.

## Patients and methods

The study cohort was composed of tumor samples referred to the Laboratory of Translational Oncology (LAOT) at the Gregorio Marañón University Hospital (HGUGM) for Prosigna^®^ assay testing between 2014 and 2020. The LAOT is a credited laboratory for the Prosigna^®^ test that provides this service to several hospitals in Spain, Portugal, Latin America, and the Middle East. The Prosigna^®^ test was performed in patients with early HR+/HER2+ breast cancer.

For this test, RNA was isolated from formalin-fixed paraffin-embedded breast tumor tissues. A pathologist examined a hematoxylin and eosin (H&E)-stained slide and identified (and marked) the area of invasive breast carcinoma suitable for the test. The pathologist also measured the tumor surface area, which determined the number of unstained slides required for the test, and tumor cellularity to ensure the presence of sufficient tumor tissue for the test. A trained technician macrodissected the area on the unstained slides corresponding to the tumoral area on the H&E-stained slide and isolated RNA from the tissue. The isolated RNA was subsequently run on a NanoString nCounter Dx Analysis System for providing the test results. Patients with BC whose samples were used in the Prosigna^®^ platform signed an informed consent form. This consent included an agreement to permit comparison of their clinical and genomic data provided by the Prosigna^®^ test for identifying a pattern of clinical variables which, together with the Risk of Recurrence (ROR), could provide information for stratifying the patients. This study (Code GOM-HGUGM-2019-06) was approved by the corresponding regulatory authorities and complies with the REMARK recommendations [11].

Patients were classified as luminal A, luminal B, basal like, or HER2 enriched using nCOUNTER/ Prosigna^®^ (https://www.prosigna.com/en-gb/). Data on the ER, PR, and Ki67 (reported as the proportion of stained cells for all three markers) were determined at local laboratories, according to the ASCO/CAP recommendations [12], and collected in the database. All tumors were required to be HER2 negative as per local evaluation, according to the applicable international practice guidelines. To assess the accuracy of three different surrogate molecular classifications (referred to as “proxy classifications” and specified in Table 1) to predict the real intrinsic subtypes as determined by the Prosigna^®^ test [5–7], tumors were classified based on the clinicopathological information compiled in the requisition form and thereafter compared with the Prosigna^®^ subtype taken as the “gold standard”. For those patients who do not meet the requirements of every proxy and remain unclassified by at least one proxy, characteristics were analyzed between them to test if some bias could be introduced by this unbalance. Numerical variables were tested with Wilcoxon Rank Sum test and categorical variables were tested with Fisher’s exact test.

**Table 1:** Three proxy intrinsic subtype classifications according to the clinicopathological surrogates by Cheang [5], Prat [6], and Maissonneuve [7]

PROXY 1 by Cheang et al. [5]	Luminal A: ER and/ or PR positive, HER2 negative, Ki67 low (Ki67 < 14%)
	Luminal B: ER and/ or PR positive, HER2 negative, and Ki67 high (Ki67 ≥ 14%)
PROXY 2 by Prat et al. [6]	Luminal A: ER positive/HER2 negative, PR > 20%, Ki67 < 14%
	Luminal B: ER positive/HER2 negative/Ki67 < 14%/PR ≤ 0% or ER positive/HER2 negative/Ki67 > 14%
PROXY 3 by Maissonneuve et al. [7]	Luminal A like: ER positive, HER2 negative, and at least one of the following conditions:
	*Ki67 low expression (< 14%) or
	*Ki67 intermediate expression (14–19%) and PR high expression (≥ 20%)
	Luminal B like: HER2 negative, ER positive, and at least one of the following conditions:
	*Ki67 intermediate expression (14–19%) and PR negative or low expression (< 20%) or
	*Ki67 high expression (≥ 20%)

For the secondary objective of our study, we performed a receiver operating characteristic (ROC) analysis to identify the Ki67 cut-off that more accurately predicts the segregation into luminal A (LumA) and luminal B (LumB) Prosigna^®^ subtypes in the population of patients with luminal tumors, selecting that cut-off that maximizes Youden’s J. Finally, we compared the surrogate classifications with the ROR (risk of relapse score) by Prosigna^®^ to assess the accuracy of the therapeutic decision based on these three “proxy classificators” versus Prosigna^®^. Hence, we included only node-negative BC patients with a luminal subtype according to Prosigna^®^, assuming that patients with LumA and LumB tumors assessed by immunohistochemistry (IHC) were to be treated using with sole endocrine therapy and chemoendocrine therapy, respectively. Similarly, we assumed that patients with low ROR (0–40) were treated with endocrine therapy alone, while those with high and intermediate ROR (60–100) were treated with chemoendocrine therapy.

The parameters provided by the Prosigna^®^ test (ROR and intrinsic subtype) were correlated with the clinical variables to determine the degree of association of two or more variables. The accuracy, sensitivity, and specificity were determined using R version 4.2.1 [13]. Cohen’s Kappa was computed using the vcd package in R [14]. Sankey diagrams were plotted on an online platform (https://www.sankeymatic.com) and ROC diagrams were obtained using GraphPad version 5.

## Results

A total of 1104 breast tumor samples of patients from eight countries (Online Resource 1: Supplementary Fig. 1) were processed and analyzed (Fig. 1, CONSORT diagram) from 2014 to 2020 in the HGUGM reference laboratory. Of these, 1049 had data regarding the Ki67 index for the primary analysis.

**Fig. 2:**
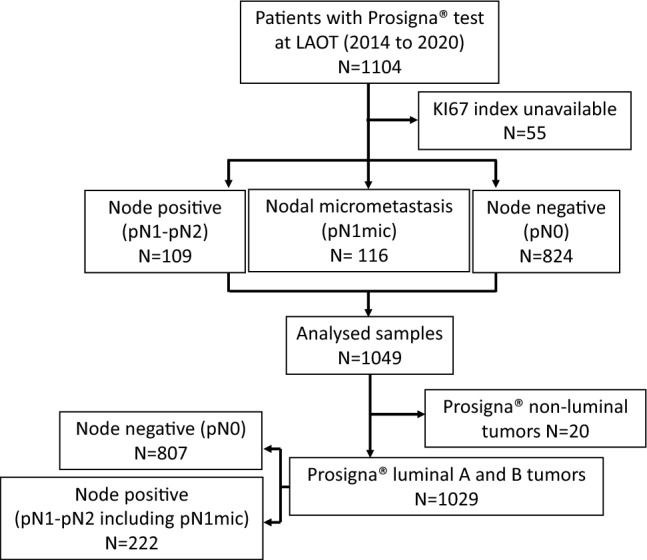
Consort diagram (*LAOT* Laboratory of Translational Oncology)

In the overall population (*n* = 1049), 65% and 33% of the patients were classified as LumA and LumB, respectively, by Prosigna^®^, whereas 20 patients (1.9%) were classified as non-luminal (eight HER2 enriched, 12 basal like). The median age at diagnosis was 56 years (range: 22–85), and most of the patients (28%) were above 50 years (68% and 77% of the patients with LumA and LumB tumors, respectively). The series was enriched in node-negative BC (83%) according to the recommendations for the use of genomic testing in BC at each time point; only 17% of the tumors had node involvement of up to three nodes (pN1). Ductal carcinoma was the most common histologic subtype in our series (79%), and infiltrating lobular carcinomas were more frequently characterized as LumA (16%) than LumB (8%). The distribution of the different pathologic markers (ER, PR, and Ki67) used in routine practice are summarized in Table 2.

**Table 2:** Tumor and patient characteristics of the analyzed samples

*n* = 1049	Prosigna^®^ molecular subtype							
	Luminal A		Luminal B		HER2-enriched		Basal-like	
	*n* = 679	%	*n* = 350	%	*n* = 8	%	*n* = 12	%
Age at diagnosis (years)								
< 50	211	31	74	21	3	38	5	42
> 50	459	68	271	77	5	63	7	58
Na	9	1	5	1	0	0	0	0
Tumor type								
Ductal	522	77	293	83	7	88	11	92
Lobular	110	16	29	8	0	0	0	0
Others	31	5	19	5	0	0	1	8
Na	16	2	9	3	1	13	0	0
Tumor size (mm)								
≤ 20	536	79	234	67	6	75	10	83
> 20	143	21	116	33	2	25	2	17
ER								
Positive	676	100	350	100	8	100	8	67
Negative	1	0	0	0	0	0	4	33
Na	2	0	0	0	0	0	0	0
PR								
Positive	611	90	324	92	5	63	8	67
Negative	66	10	26	7	3	38	4	33
Na	2	0	0	0	0	0	0	0
Ki67 index (%)								
< 14	399	59	69	20	0	0	0	0
14–20	189	28	119	34	1	13	0	0
> 20	91	13	162	46	7	88	12	100
Ki67 index (%)								
< 13	392	58	64	18	0	0	0	0
> 13	287	42	286	81	8	100	12	100
Histological grade								
1	81	12	12	3	0	0	0	0
2	379	56	177	50	1	13	2	17
3	25	4	53	15	2	25	6	50
Na	194	29	109	31	5	63	4	33
Prosigna ROR								
Low	461	68	1	0	0	0	1	8
Intermediate	194	29	145	41	1	13	9	75
High	24	4	204	58	7	88	2	17
Lymph nodes								
0	521	77	286	82	7	88	10	83
1–3	158	23	64	18	1	13	2	17

*NA* non-available, *ER* estrogen receptor, *PR* progesterone receptor, *HER* human epidermal growth factor receptor 2, *ROR* risk of recurrence

In the overall population, 1029 of the 1049 patients were classified as LumA (65%) or LumB (33%) by Prosigna^®^, while 20 patients (1.9%) were classified as non-luminal.

Correlation analysis (IHC vs. Prosigna^®^) was performed only in the population of 1029 patients with luminal tumors according to Prosigna^®^. The luminal population was representative of the entire series based on the main clinical characteristics, and no major differences were observed. Differences in age, Prosigna ROR, tumor size, tumor type, histological grade, and node status were tested between samples classified by every proxy and samples unclassified by at least one proxy. A significant difference was found only in nodal status (37% of unclassified patients were node positive, vs 21% of classified patients, *p* value = 0.016).

The agreement between the Prosigna^®^ subtype and the three proxy classifications is presented in Table 3 and Online Resource: Supplementary Fig. 3. Not all the clinicopathological variables were available for all the samples, thus explaining the slight imbalance between the sample sizes in the different proxies. The concordance with Prosigna^®^ for each surrogate classification was mostly poor, Proxy 1 (*k* = 0.34, 95% confidence interval [CI] 0.288–0.390), Proxy 2 (*k* = 0.27, 95% CI 0.221–0.315), and Proxy 3 (*k* = 0.37, 95% CI 0.311–0.427), with an accuracy slightly greater than 0.6. The Proxy 3 classification by Maisonneuve et al. [7] demonstrated the best concordance; however, the kappa index was still below 0.4 in the three of them.

**Table 3:** Concordance between Prosigna^®^ assay and IHC classifications: Proxy 1, Proxy 2, and Proxy 3 [5–7]

Proxy 1 (*n* = 1028)	Prosigna^®^						
	LumA	LumB					
LumA	391 (38%)	65 (6%)	Kappa	95% CI	Accuracy	TPR	TNR
LumB	287 (28%)	285 (28%)	0.34	(0.288–0.390)	0.66	0.58	0.81

Proxy 2 (*n* = 990)	Prosigna^®^						
	LumA	LumB					
LumA	300 (30%)	45 (5%)	Kappa	95% CI	Accuracy	TPR	TNR
LumB	352 (36%)	293 (30%)	0.27	(0.221–0.315)	0.59	0.46	0.87

Proxy 3 (*n* = 1020)	Prosigna^®^						
	LumA	LumB					
LumA	490 (49%)	117 (11%)	Kappa	95% CI	Accuracy	TPR	TNR
LumB	185 (18%)	228 (22%)	0.37	(0.311–0.427)	0.70	0.73	0.66

*IHC* immunohistochemistry, *CI* confidence interval, *LumA* luminal A, *LumB* luminal B, *TPR* true positive rate, *TNR* true negative rate

Each classifier exhibited a different misclassification pattern. Proxy 3 had the best accuracy of the three tested surrogates (0.7), with moderately better sensitivity (0.73) than specificity (0.66). With this classifier, 18% of LumB tumors were misclassified. Proxy 1 had low sensitivity (0.58) and high specificity (0.81), indicating that it prioritizes the detection of LumB tumors at the cost of misclassifying 28% of the LumA tumors. Proxy 2 followed a more intense version of this pattern, with lower sensitivity (0.46) and higher specificity (87); 36% of the LumA tumors were misclassified.

One of the secondary objectives of our trial was to determine the Ki67 cut-off value that more accurately distinguishes between the LumA and LumB subtypes (Prosigna^®^). We analyzed all the 1029 patients with Prosigna^®^ luminal tumors.

**Fig. 4:**
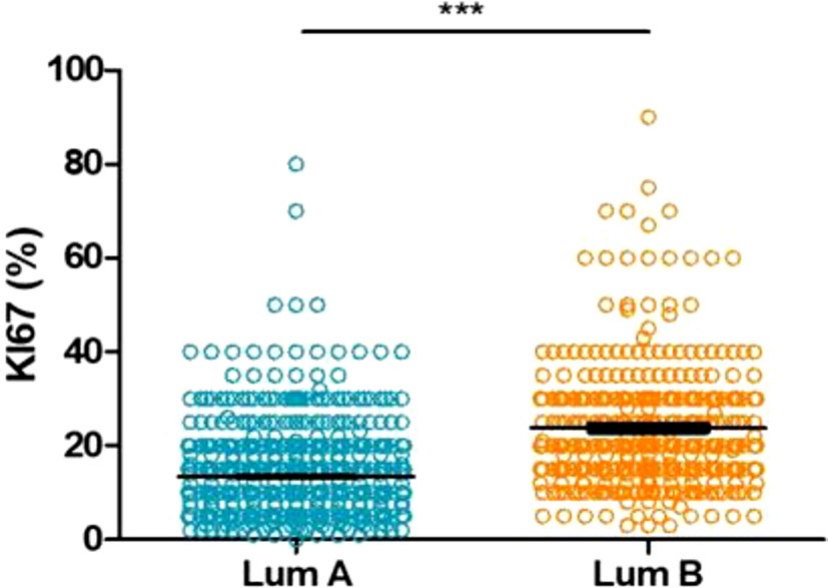
Ki67 distribution in the luminal tumor samples (*n* = 1029)

**Fig. 5:**
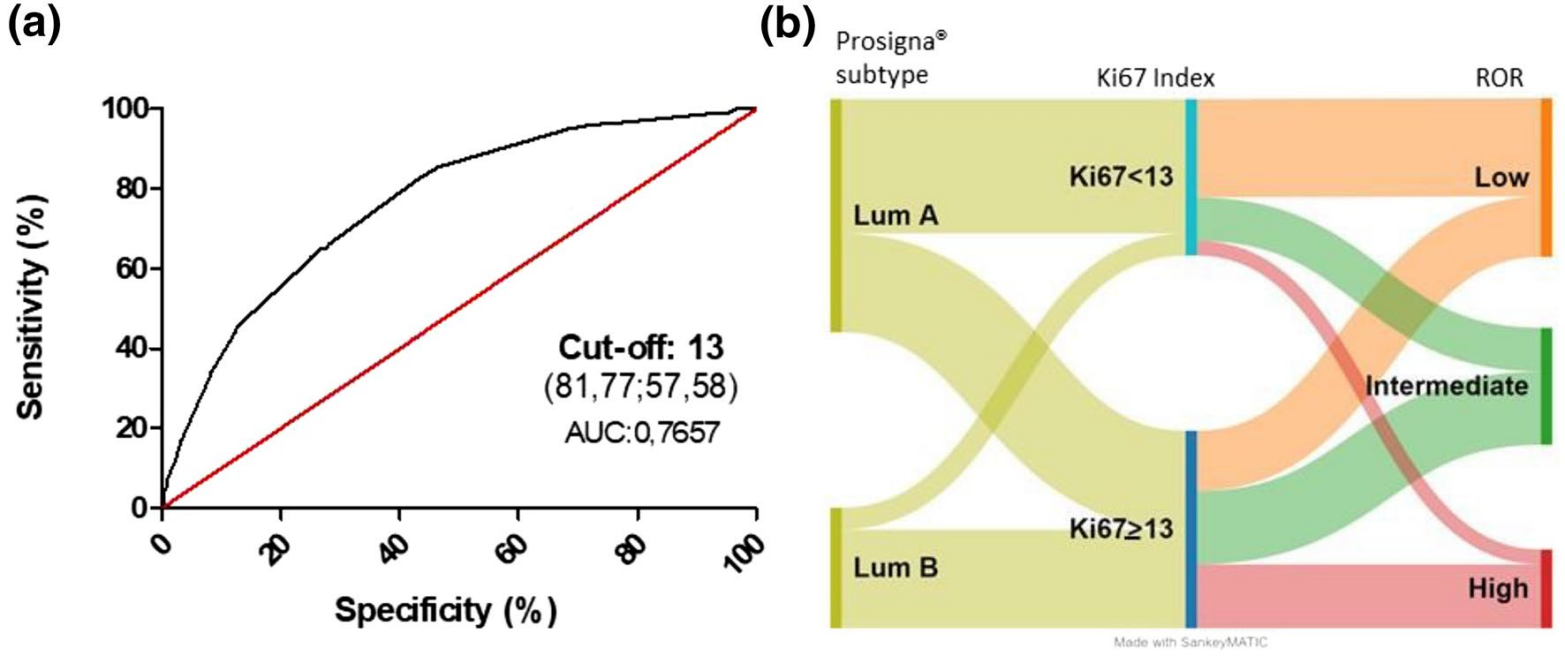
**a** ROC curve of Ki67 in the luminal samples (Prosigna^®^); **b** Sankey diagram for the Prosigna.^®^ subtypes vs. Ki67 index (cut-off 13%) and risk of recurrence (*ROC* receiver operating characteristic, *LumA* luminal A, *LumB* luminal B)

The distribution of Ki67 proliferation markers is depicted in Fig. 4, with median Ki67 levels of 10% (range 0–80) and 20% (range 3–90) in the LumA and LumB tumors, respectively. The best Ki67 cut-off was 13% (Fig. 5) according to the ROC analysis (area under the curve 0.7657; 95% CI 0.7360–0.7955; *p* < *0.0001*). In total, 55.8% (*n* = 574) and 44.2% (*n* = 455) of the tumors had high and low Ki67, respectively, according to this threshold. The rate of LumA Prosigna^®^ tumors with low Ki67 was 58%, whereas 82% of LumB tumors had high Ki67.

Table 4 and Online Resource: Supplementary Fig. 6 depict the correlation between the ROR and IHC classifications in node-negative patients with luminal tumors (807 of the 1029 samples by Prosigna^®^, resulting in 807, 782, and 802 samples for Proxy 1, 2, and 3, respectively). The concordance between the three classifications and ROR was low. A specific analysis in luminal node-negative patients aged 50 or older (*n* = 571) has been performed without relevant differences in comparison to the previous results (see Online Resource: Supplementary Table 5).

**Table 4:** Concordance between the Prosigna^®^ assay and IHC classifications according to the risk of recurrence (ROR): (a) low ROR (0–40) and (b) intermediate (41–60) + high (61–100) ROR

Proxy 1 (*n* = 407)	Low ROR						
	LumA	LumB					
LumA	247 (61%)	0 (0%)	Kappa	95% CI	Accuracy	Sensitivity	Specificity
LumB	159 (39%)	1 (0%)	0.008	(-0.007 to 0.022)	0.61	0.61	1.00

Proxy 2 (*n* = 394)	Low ROR						
	LumA	LumB					
LumA	186 (47%)	0 (0%)	Kappa	95% CI	Accuracy	Sensitivity	Specificity
LumB	207 (53%)	1 (0%)	0.005	(-0.004 to 0.013)	0.47	0.47	1.00

Proxy 3 (*n* = 406)	Low ROR						
	LumA	LumB					
LumA	307 (76%)	1 (0%)	Kappa	95% CI	Accuracy	Sensitivity	Specificity
LumB	98 (24%)	0 (0%)	-0.005	(-0.014 to 0.005)	0.76	0.76	0.00

Proxy 1 (*n* = 400)	Intermediate + high ROR						
	LumA	LumB					
LumA	43 (11%)	52 (13%)	Kappa	95% CI	Accuracy	Sensitivity	Specificity
LumB	72 (18%)	233 (58%)	0.202	(0.099 to 0.305)	0.69	0.37	0.82

Proxy 2 (*n* = 388)	Intermediate + high ROR						
	LumA	LumB					
LumA	37 (10%)	36 (9%)	Kappa	95% CI	Accuracy	Sensitivity	Specificity
LumB	75 (19%)	240 (62%)	0.223	(0.119 to 0.327)	0.71	0.33	0.87

Proxy 3 (*n* = 396)	Intermediate + high ROR						
	LumA	LumB					
LumA	60 (15%)	96 (24%)	Kappa	95% CI	Accuracy	Sensitivity	Specificity
LumB	55 (14%)	185 (47%)	0.163	(0.066 to 0.26)	0.62	0.52	0.66

*IHC* immunohistochemistry, *CI* confidence interval, *LumA* luminal A, *LumB* luminal B

According to the International Ki67 Working Group (IKWG) recommendations, Ki67 can be categorized into low (≤ 5%), intermediate (6–29%), and high (≥ 30%) [15]. Therefore, the Ki67 effect on ROR categorization by Prosigna^®^ in the node-negative luminal subgroup of patients was analyzed (Online Resource: Supplementary Fig. 7). The Ki67 low-risk index encompassed 137 patients, among whom 82% had a low ROR vs. 12% with an intermediate ROR and 6% with a high ROR. The Ki67 intermediate-risk group (*n* = 528) included 51% of the patients with low ROR vs. 34% with intermediate and 16% with high ROR. Finally, of the 142 patients included in the KI67 high-risk group, 18%, 39%, and 43% had low, intermediate, and high ROR, respectively.

In the overall population, a significant agreement (95% CI 0.1261–0.2146) was observed between Ki67 and ROR (*κ* = 0.1703; *p* < *0.001*).

None of the individual-risk Ki67 groups showed significant agreement between Ki67 and ROR; the intermediate-risk Ki67 group was less significant (low-risk Ki67 group: 95% CI − 1.208 to 1.64; *κ* = 0.2164; *p* = 0.7658; high-risk Ki67 group: 95% CI − 2.028 to 2.602; *κ* = 0.2871; *p* = 0.8079; and intermediate-risk Ki67 group: 95% CI − 1.438 to 1.572; *κ* = 0.0669; *p* = 0.9306, respectively).

With a continuous distribution of Ki67 and ROR among low-risk Ki67 patients, the Spearman correlation coefficient was -0.009 (*p* = 0.9136). Among the intermediate-risk and high-risk Ki67 patients, the Spearman correlation coefficient was 0.329 (*p* < 0.001) and 0.198 (*p* = 0.018), respectively.

## Discussion

This population-based study included patients with HR+/HER2− BC whose physicians had reservations concerning the risk of relapse (and consequently, the use of chemotherapy in addition to endocrine therapy) and, therefore, required to perform the Prosigna^®^ test for aiding in the decision-making process. In this series, we demonstrated poor agreement between the three different surrogate definitions of the luminal and genomic subtypes using the Prosigna^®^_PAM50 algorithm.

We considered three different surrogate classifications in our analysis to ensure a broader analysis and define the best approach to be implemented in the clinical practice setting. In 2011 and further refined in 2013 [16], the St. Gallen consensus panel [16, 17] included this consideration as the basis for their clinical recommendations evolving from the classic 3-pathological biomarker approach (ER, PR, and HER2), which divided BC into luminal, HER2, and triple-negative subtypes [18], into a four-category classification that considered the Ki67 index as the fourth potential biomarker and aimed to subdivide luminal tumors into luminal A and B. Cheang and Prat classifications [5, 6] guided the definitions from the panel. The current ESMO clinical practice guidelines [9] and the latest St. Gallen consensus for the treatment of early BC [10] follow this initial approach. Maisonneuve et al. [7] attempted to validate the previous surrogate definitions of intrinsic BC subtypes in a large Italian series with long-term follow-up and proposed new surrogate definitions using the same four IHC biomarkers that maximized the detection of luminal A tumors. However, more recent attempts have been made to provide better surrogate classifications by including other pathologic characteristics in the definitions. Lundgren et al. [19] included histological grade in the model based on the prognostic value of this biomarker and their validation in a SCAN-B project suggested that the combination of histologic grade and Ki67 could identify molecular luminal A tumors, especially when other clinicopathological factors were identified similarly. Other groups have also tested the role of histological grade as a variable for approaching the molecular subtype from a predictive perspective to chemotherapy in the neoadjuvant setting [20] supporting interest in this marker. More recently, Hold et al. [21] developed the Prolif surrogate classifier [22] and tested its performance in a series of patients that were included in two clinical trials with long-term follow-up [21, 22]. The study confirmed the limited ability of all surrogate classifiers in differentiating between luminal A and B intrinsic subtypes with different patterns of misclassification based on the combination of biomarkers.

This yields a discussion regarding the role of proliferation markers and Ki67, particularly as part of the surrogate, in approaching the intrinsic BC subtypes. Proliferation is a molecular characteristic that relies on the differential biological behavior of luminal A and B. Interestingly, endocrine receptor and proliferation genes are commonly found in the different prognostic signatures of BC [23], while the derived recurrence scores provided by commercially available genomic platforms are differentially driven by each of them [24]. Proliferation can be measured through the mRNA levels of different genes (proliferation signature modules) or the assessment of protein levels using IHC. Ki67, a measure of tumor proliferation by IHC, is usually part of the pathology report of a breast tumor in the clinical practice setting. However, many efforts have been undertaken to address methodological issues in the validation of this currently used biomarker, which has been used not only to estimate prognosis in early-stage disease but also to predict the potential utility of chemotherapy and monitor patients selected for primary systemic strategies [15]. This controversy has escalated recently with the approval of adjuvant abemaciclib by the FDA for patients with high-risk HR + HER2 + BC based on the Ki67 selection criteria [25], highlighting reproducibility as one of the limitations when Ki67 is used on a widespread basis. In fact, the expanded adjuvant indication of abemaciclib (March 2023) removes the Ki-67 score requirement for patient selection. Ki67 is a continuous variable and its optimal threshold has not been completely established. The lack of universal standardization of this biomarker in clinical practice could partially justify the poor performance of BC surrogates in real-world settings, as seen in our series and suggested by others [19]. Several sources of variability (pre-, post-, and analytical) should be considered in pathology owing to the lack of standardization; thus, proxy classifiers based on the local pathology report were employed in our series.

A recent retrospective study demonstrated an insignificant correlation between Ki67 (determined according to specific international recommendations [IKWG] and the Oncotype Dx recurrence score [RS]) [26], questioning the utility of Ki67 as a surrogate for RS to guide therapeutic decisions in clinical practice, especially for the low and intermediate range of the Ki67 index [26]. In our series, the correlation of Ki67 did not follow the same tendency, suggesting that low-risk Ki67 categorization could be useful to define the Prosigna^®^ ROR; however, it remained insignificant by itself. Furthermore, when we correlated the three different IHC-based surrogates with the Prosigna^®^ ROR, the concordance remained low. Therefore, molecular taxonomy adds information to the classic pathologic classification of BC and using appropriate terminology when referring to the classifier for defining the tumor subtype both at the research level and in daily clinical practice is crucial [27, 28].

Our analysis of the database of a centralized credited reference laboratory to determine the intrinsic subtype by the gold standard Prosigna^®^ commercial assay has some strengths. The study compiles data from more than 1000 samples from patients distributed worldwide, mainly from southern Europe, treated in a real-world setting. In 2012, the IMPAKT Working Group Consensus Statement [29] evaluated the evidence on genomic tests in BC and encouraged the creation of registries for patients where genomic testing was performed in daily practice. In line with this recommendation, we contribute the experience of our laboratory in a contemporary time period of six years. However, we must recognize several limitations. While molecular profiling was performed in a centralized laboratory, the IHC information needed to calculate each proxy surrogate was obtained according to local protocols valid in each institution at the time of testing; thus, centralized staining for IHC markers could not be performed. The intrinsic retrospective nature of this series, which focuses on samples from different parts of the world with no long-term survival data, precludes our ability to correlate the ROR data and survival in this patient cohort.

The most commonly used IHC surrogate in clinical practice is the 3-IHC surrogate based on ER, PR, and HER2, which divides invasive BC into luminal, HER2, and triple-negative subtypes. The St. Gallen panel adopted 4/5-IHC surrogates [5, 6], as previously discussed, and focused on their ability to distinguish between patients with HR+/HER2+ tumors who could benefit from systemic adjuvant chemotherapy. Therefore, the aim was to avoid undertreatment by prioritizing the sensitivity for luminal B detection, as demonstrated in our analysis. Newer refined surrogates [7] were better at identifying luminal A tumors than potential candidates for chemotherapy de-escalation, as also reported in our series, which is significantly enriched in luminal A tumors. For all surrogate models, a critical common limitation is the lack of standardization in all biomarker determinations and a uniform cut-off.

## Conclusion

In our study, the concordance between luminal subtypes determined using three different IHC-based classifiers and the nCOUNTER PAM50 assay was clearly suboptimal. Between 5 and 12% of the nCOUNTER PAM50 luminal B tumors were classified as luminal A by IHC and could be undertreated. Conversely, 19–36% of nCOUNTER PAM50 luminal A tumors were classified as luminal B by IHC, which could receive unnecessary chemotherapy. This limitation should be considered when personalized oncology paradigm is translated into a clinical practice setting.

### Supplementary Information

Below is the link to the electronic supplementary material.

Supplementary file1 (DOCX 578 kb)

## Data Availability

The datasets that support the findings of this study are subject to third-party restrictions owing to contractual agreements, and they will be made available upon reasonable request. Data access requests were subject to approval and should be addressed to Dr. Martin (https;//mmartin@geicam.org).

## Abbreviations

BC: Breast cancer
HR: Hormone receptor
HER2: Human epidermal growth factor receptor 2
ER: Estrogen receptor
PR: Progesterone receptor
H&E: Hematoxylin and eosin
ROR: Risk of recurrence
ROC: Receiver operating characteristic
IHC: Immunohistochemistry
RS: Recurrence score
IKWG: International Ki67 Working Group

## Declarations

### Clinical trial information

This study was evaluated and approved by the corresponding Spanish Agency for Medicines and Sanitary Products (AEMPS) on January 16, 2020, in accordance with the ethical standards of the 1964 Declaration of Helsinki and its later amendments.
