# Supplementary material for: Correlation between breast cancer subtypes determined by immunohistochemistry and n-COUNTER PAM50 assay: a real-world study
Source: Breast Cancer Res Treat. 2023 Sep 29;203(1):163–72. doi: 10.1007/s10549-023-07094-9 (PMC10771357; doi:10.1007/s10549-023-07094-9)

# **SUPPLEMENTARY MATERIAL**

Journal name: *Breast Cancer Research and Treatment*

CORRELATION BETWEEN BREAST CANCER SUBTYPES DETERMINED BY IMMUNOHISTOCHEMISTRY AND N-COUNTER PAM-50 ASSAY: A REAL-WORLD STUDY

# **Authors**

Sara. Lopez-Tarruella^1^, María. Del Monte-Millán^2^, Marta. Roche-Molina^3^, Yolanda. Jerez^2^, Isabel. Echavarria Diaz-Guardamino^2^, Blanca. Herrero Lopez^3^, Salvador. Gamez Casado^3^, Iván. Marquez-Rodas^2^, Enrique. Alvarez^3^, María. Cebollero^4^, Tatiana. Massarrah^2^, Inmaculada. Ocaña^3^, Ainhoa. Arias^3^, José Ángel García Saenz^5^, Fernando Moreno Anton^5^, Clara Olier Garate^6^, Diana Moreno Muñoz^6^, David. Marrupe Gonzalez^7^, Miguel.Ángel Lara Alvarez^8^, Santos Enrech^9^, Coralia Bueno Muiño^10^, Miguel Martín^1*^

# **Authors’ affiliations**

^1^ Medical Oncology Department, Hospital General Universitario Gregorio Marañón, Instituto de Investigación Sanitaria Gregorio Marañon (IiSGM), CIBERONC, Geicam, Universidad Complutense, 28007 Madrid, Spain

^2^ Medical Oncology Department, Hospital General Universitario Gregorio Marañón, Instituto de Investigación Sanitaria Gregorio Marañón (IiSGM), CiberOnc, Madrid, Spain

^3^ Medical Oncology Department, Hospital General Universitario Gregorio Marañón Instituto de Investigación Sanitaria Gregorio Marañón (IiSGM), Madrid, Spain

^4^ Pathology Service, Hospital General Universitario Gregorio Marañón, Madrid, Spain

^5^ Medical Oncology Department, Hospital Clínico San Carlos, Instituto de Investigación Sanitaria San Carlos (IdISSC), CIBERONC, Madrid, Spain

^6^ Medical Oncology Department, Hospital Universitario Fundación Alcorcón, Alcorcon, Spain,

^7^Dept. Oncologia, Hospital Universitario de Móstoles, Mostoles, Spain,

^8^ Medical Oncology Department, Hospital Universitario Infanta Leonor, Universidad Complutense, Madrid, Spain

^9^ Medical Oncology Department, Hospital Universitario de Getafe, Madrid, Spain

^10^ Medical Oncology Department, Hospital Infanta Cristina (Parla), Fundación de Investigación Biomédica del H.U. Puerta de Hierro, Majadahonda, 28009 Madrid, Spain

*Corresponding author; [mmartin@geicam.org](mailto:mmartin@geicam.org)

**Supplementary Fig. 1** Number of Prosigna® tests performed by country and centralized from 2014 to 2020

**Supplementary Fig. 3** Sankey graphs for each proxy correlation and the intrinsic subtype in the Luminal population **a**) Proxy 1: Cheang; **b**) Proxy 2: Prat and **c**) Proxy 3: Maisonneuve [5, 6, 7] (LumA, luminal A; LumB, luminal B; IHC, immunohistochemistry).


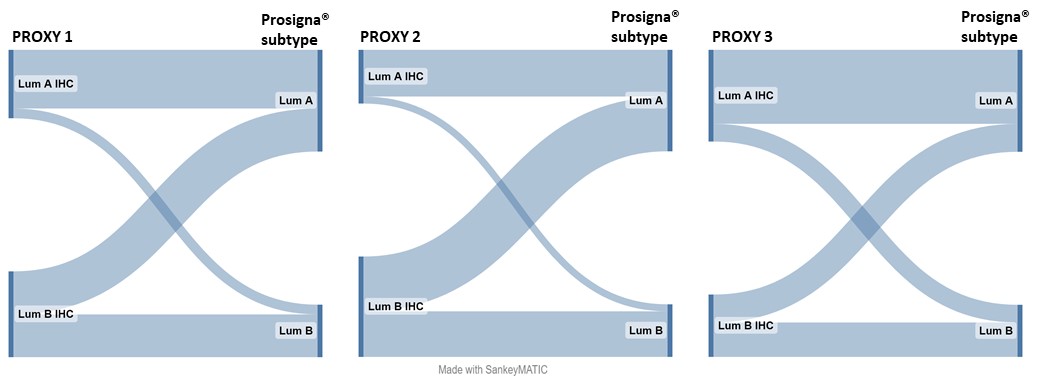


**Supplementary Fig. 6** Sankey graphs for each proxy subtype correlation and the Prosigna® ROR in luminal tumors in node-negative patients **a**) Proxy 1: Cheang; **b**) Proxy 2: Prat and **c**) Proxy 3: Maisonneuve [5, 6, 7] (LumA, luminal A; LumB, luminal B; ROR, Risk of Recurrence).


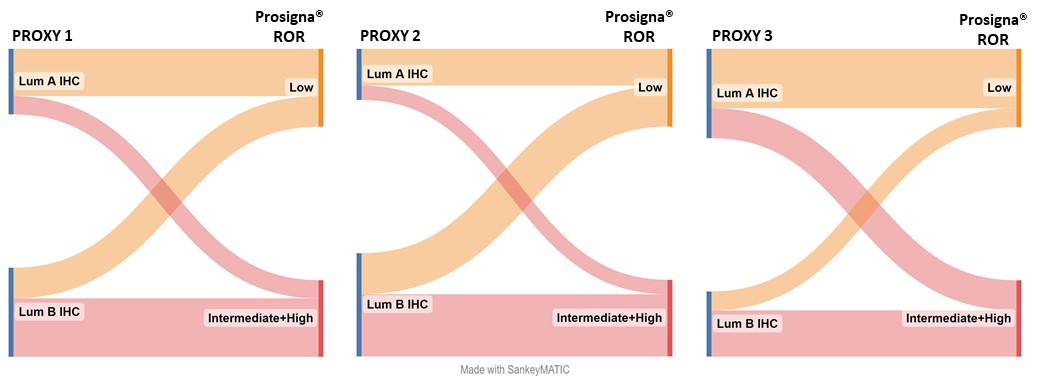


**Supplementary Table 5**. (Suppl material) Concordance between Prosigna® assay and IHC classifications depending on the risk of recurrence (ROR): a) Low ROR (0-40); b) Intermediate (41-60) + High ROR (61-100) in node –negative Luminal patients aged 50 or older.

| Proxy 1 (n=275) | **LOW ROR** | |  |  |  |  |  |
| --- | --- | --- | --- | --- | --- | --- | --- |
|  | Lum A | Lum B |  |  |  |  |  |
| Lum A | 165 (60%) | 0 (0%) | Kappa | 95% CI | Accuracy | Sensitivity | Specificity |
| Lum B | 109 (40%) | 1 (0%) | 0,011 | (-0.010 – 0.032) | 0,60 | 0,60 | 1,00 |
|  |  |  |  |  |  |  |  |
| Proxy 2 (n=264) | **LOW ROR** | |  |  |  |  |  |
|  | Lum A | Lum B |  |  |  |  |  |
| Lum A | 116 (44%) | 0 (0%) | Kappa | 95% CI | Accuracy | Sensitivity | Specificity |
| Lum B | 147 (56%) | 1 (0%) | 0,006 | (-0.005 – 0.017) | 0,45 | 0,45 | 1,00 |
|  |  |  |  |  |  |  |  |
| Proxy 3 (n=275) | **LOW ROR** | |  |  |  |  |  |
|  | Lum A | Lum B |  |  |  |  |  |
| Lum A | 203 (74%) | 1 (0%) | Kappa | 95% CI | Accuracy | Sensitivity | Specificity |
| Lum B | 71 (26%) | 0 (0%) | -0,007 | (-0.020 – 0.006) | 0,75 | 0,75 | - |
|  |  |  |  |  |  |  |  |
| Proxy 1 (n=306) | **INTERMEDIATE+HIGH ROR** | |  |  |  |  |  |
|  | Lum A | Lum B |  |  |  |  |  |
| Lum A | 35 (11%) | 42 (14%) | Kappa | 95% CI | Accuracy | Sensitivity | Specificity |
| Lum B | 47 (15%) | 182 (59%) | 0,244 | (0.125 – 0.363) | 0,71 | 0,43 | 0,81 |
|  |  |  |  |  |  |  |  |
| Proxy 2 (n=297) | **INTERMEDIATE+HIGH ROR** | |  |  |  |  |  |
|  | Lum A | Lum B |  |  |  |  |  |
| Lum A | 30 (10%) | 31 (10%) | Kappa | 95% CI | Accuracy | Sensitivity | Specificity |
| Lum B | 50 (17%) | 186 (63%) | 0,265 | (0.145 – 0.384) | 0,73 | 0,38 | 0,86 |
|  |  |  |  |  |  |  |  |
| Proxy 3 (n=303) | **INTERMEDIATE+HIGH ROR** | |  |  |  |  |  |
|  | Lum A | Lum B |  |  |  |  |  |
| Lum A | 46 (15%) | 78 (26%) | Kappa | 95% CI | Accuracy | Sensitivity | Specificity |
| Lum B | 36 (12%) | 143 (47%) | 0,182 | (0.076 – 0.290) | 0,63 | 0,56 | 0,65 |

**Supplementary** **Fig. 7**) Prosigna® ROR distribution by Ki67 risk group in node negative luminal samples (Prosigna®) (ROR, Risk of Recurrence)


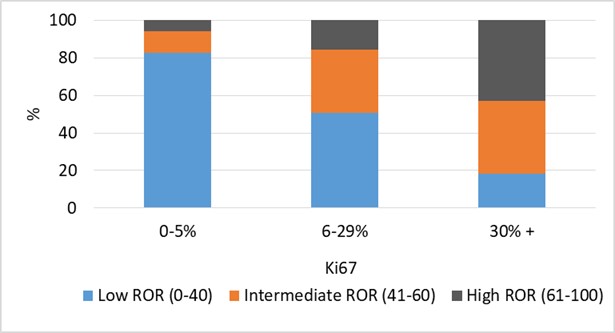

Supplement: Supplementary file 1 — Supplementary file1 (DOCX 578 kb) [file 10549_2023_7094_MOESM1_ESM.docx]
